# Peripheral Sensitization Increases Opioid Receptor Expression and Activation by Crotalphine in Rats

**DOI:** 10.1371/journal.pone.0090576

**Published:** 2014-03-04

**Authors:** Vanessa Olzon Zambelli, Ana Carolina de Oliveira Fernandes, Vanessa Pacciari Gutierrez, Julio Cesar Batista Ferreira, Carlos Amilcar Parada, Daria Mochly-Rosen, Yara Cury

**Affiliations:** 1 Laboratório Especial de Dor e Sinalização, Instituto Butantan, São Paulo, SP, Brazil; 2 Departamento de Anatomia, Instituto de Ciências Biomédicas, Universidade de São Paulo, São Paulo, SP, Brasil; 3 Departamento de Fisiologia e Biofísica, Instituto de Biociências (UNICAMP) Rua Monteiro Lobato, Cidade Universitária, Campinas, SP, Brazil; 4 Department of Chemical and Systems Biology, Stanford University, School of Medicine, Stanford, California, United States of America; The Hebrew University Medical School, Israel

## Abstract

Inflammation enhances the peripheral analgesic efficacy of opioid drugs, but the mechanisms involved in this phenomenon have not been fully elucidated. Crotalphine (CRP), a peptide that was first isolated from South American rattlesnake *C.d. terrificus* venom, induces a potent and long-lasting anti-nociceptive effect that is mediated by the activation of peripheral opioid receptors. Because the high efficacy of CRP is only observed in the presence of inflammation, we aimed to elucidate the mechanisms involved in the CRP anti-nociceptive effect induced by inflammation. Using real-time RT-PCR, western blot analysis and ELISA assays, we demonstrate that the intraplantar injection of prostaglandin E2 (PGE_2_) increases the mRNA and protein levels of the µ- and κ-opioid receptors in the dorsal root ganglia (DRG) and paw tissue of rats within 3 h of the injection. Using conformation state-sensitive antibodies that recognize activated opioid receptors, we show that PGE_2_, alone does not increase the activation of these opioid receptors but that in the presence of PGE_2_, the activation of specific opioid receptors by CRP and selective µ- and κ-opioid receptor agonists (positive controls) increases. Furthermore, PGE2 down-regulated the expression and activation of the δ-opioid receptor. CRP increased the level of activated mitogen-activated protein kinases in cultured DRG neurons, and this increase was dependent on the activation of protein kinase Cζ. This CRP effect was much more prominent when the cells were pretreated with PGE_2_. These results indicate that the expression and activation of peripheral opioid receptors by opioid-like drugs can be up- or down-regulated in the presence of an acute injury and that acute tissue injury enhances the efficacy of peripheral opioids.

## Introduction

Clinical and experimental results have shown that the peripheral analgesic efficacy of opioids is enhanced in the presence of inflammation and tissue injury [1], [2]. The mechanisms responsible for this phenomenon involves the following events: increases in the opioid receptor mRNA level and opioid receptor expression level [3]–[5], an increase in the axonal transport and accumulation of these receptors in the peripheral sensory nerve endings [6], and an increase in the opioid agonist binding to their corresponding receptor [7], [8]. Moreover, inflammation induces the release of endogenous opioid peptides [1]. Studies investigating the molecular mechanisms involved in the enhanced effects of opioid treatments in the periphery caused by inflammation mainly focus on the µ-opioid receptor and the chronic inflammatory processes [2], [5]–[7], [9]. Inflammation enhances the axonal transport of opioid receptors toward the periphery, which is preceded by an increase in opioid receptor mRNA transcription [3], [10].

Previously, we demonstrated that crotalphine (CRP), a structural analogue to a novel analgesic peptide that was first identified in the crude venom from the South American rattlesnake *Crotalus durissus terrificus* [11], induces a potent (0.008–25 µg/kg, p.o.) and long-lasting (2–5 days) anti-nociceptive effect that is mediated by the activation of the peripheral κ-opioid receptor (prostaglandin E_2_-induced hyperalgesia) and the κ- and δ- (chronic constriction injury of the rat sciatic nerve) opioid receptors [11]–[13]. The L-arginine-nitric oxide-cGMP pathway is involved in the peripheral anti-nociceptive effect of CRP [13] and opioid agonists [14]–[17]. The activation of opioid receptors regulates a variety of intracellular signaling cascades, including the PI3Kγ/AKT signaling pathway [18], as well as the activation of mitogen-activated protein kinases (MAPKs) and protein kinase C (PKC) [19]–[21].

The high efficacy and long-lasting peripheral anti-nociceptive effects of crotalphine have only been observed in the presence of inflammation, and the mechanisms involved in the enhanced effects of CRP in the presence of inflammation are currently unknown. In the present study, we used prostaglandin E_2_ (PGE_2_)-induced hyperalgesia in a rat model to characterize the anti-nociceptive effect caused by the peripheral administration of CRP and compared this response with the responses induced by select opioid receptor agonists. Furthermore, we also investigated the intracellular signaling pathways activated by CRP and the opioid receptor agonists.

## Experimental Procedures

### Animals

Male Wistar rats (170–190 g) were used in this study. The animals were housed in a temperature-controlled (21±2°C) and light-controlled (12 h/12 h light/dark cycle) environment. All of the behavioral tests were performed between 9:00 am and 4:00 pm. Standard food and water were available *ad libitum*. All of the procedures were conducted in accordance with the guidelines for the ethical use of conscious animals in pain research published by the International Association for the Study of Pain [22] and were approved by the Institutional Animal Care Committee of the Butantan Institute (CEUAIB, protocol number 386/2007).

### Drug administration

The drugs [D-Ala^2^, N-Me-Phe^4^, Gly^5^-ol]-Enkephalin acetate salt (1) (DAMGO, 5 µg/paw); [D-Pen^2,5^, p-Cl-Phe^4^]-Enkephalin (DPDPE, 20 µg/paw); (−)-*trans*-(1S,2S)-U-50488 hydrochloride hydrate (U 50,488, 10 µg/paw) [23]; Prostaglandin E_2_ (PGE_2_, 100 ng/paw) [23], [24]; D-Phe-Cys-Tyr-D-Trp-Orn-Thr-Pen-Thr amide (CTOP, 20 µg/paw) [25], [26], nor-binaltorphimine dihydrochloride (nor-BNI, 50 µg/paw) [25], [27]; N,N-diallyl-Tyr-Aib-Aib-Phe-Leu (ICI 174,864, 10 µg/paw) [25], methiodide naloxone (1 mg/Kg, subcutaneously) were used in this study [28]. These drugs were purchased from Sigma-Aldrich, Saint Louis, MO, USA. The doses of the agonists and antagonists used in this study were determined based on the doses used in previous studies and preliminary studies [25].The peptide ζ-pseudosubstrate ([C]SIYRRGARRWRKLYRAN; amino acids 105–121 in ζ-PKC) was synthesized at the peptide and nucleotide facility at Stanford and fused to the cell permeable Tat protein transduction domain peptide. The peptide CRP (<E-F-S-P-E-N-C-Q-G-E-S-Q-P-C, where <E is pyroglutamic acid and a disulfide bond is located between 7C-14C) was synthesized by the American Peptide Co, Sunyvalle, CA, USA (product number 331065, lot number U07122A1, 98% purity, M.W. 1534,6 Daltons) using Fmoc chemistry in the solid phase, as described by Konno et al. (2008) [11]. Crotalphine (CRP) was administered using a dose of 0.6 ng/paw. The peptide was diluted in sterile saline, and the opioid agonists and antagonists were diluted in water or saline. All of the drugs were administered in a final volume of 50 µl by intraplantar (i.pl.) injection.

PGE2 was injected into the hind paw to induce hyperalgesia. CRP and the opioid agonists and antagonists were injected 2 h after the PGE2 injection. Control animals were given the same volume of sterile saline or water by i.pl. injection and were tested following the same schedule described below.

### PGE2-induced hyperalgesia

Sterile saline containing PGE2 (100 ng/paw) was injected into both hind paws for the behavioral tests and into one hind paw for the biochemical experiments. A stock solution of PGE2 was prepared by dissolving 0.5 mg of PGE2 in 1 ml of absolute ethanol. For injection into the rat paw, an aliquot of this stock solution was diluted in sterile saline. The percentage of ethanol in the solution injected into the hind paw was less than 0.1%. The pain threshold was measured before and 3 h after PGE2 injection [25], [29].

The rat paw pressure test [30] was used to evaluate hyperalgesia. An Ugo-Basile pressure apparatus was used to determine the pressure pain threshold before and 3 h after PGE2 injection. The testing was conducted blind to the group designation. Briefly, an increasing amount of force (in g, 16 g/s) was applied to the hind paw. The force needed to induce paw withdrawal was recorded as the pain threshold. To reduce the stress level, the rats were acclimated to the testing procedure 1–3 days before the experiment.

### Biochemical studies

For this study, ipsilateral and contralateral dorsal root ganglia (DRG, L4-6, pooled 7–10 ganglia) and plantar tissue were collected 3 h after the intraplantar injection of PGE2. To obtain the plantar tissue, two longitudinal incisions were made through the skin and muscle of the plantar aspect of the foot, from the proximal edge of the heel to the toes, using a blade. The skin was removed, and the adjacent subcutaneous tissue, including tendon, was collected. Nnaïvenaïve animals were used as controls.

#### Reverse Transcriptase/Real-Time Polymerase Chain Reaction

RNA was isolated from the DRGs and plantar tissue using Trizol reagent (Gibco Life Technologies, Grand Island, NY, USA). The RNA concentration and integrity were determined, cDNA was synthesized using Superscript III RNase H-Reverse Transcriptase (Invitrogen Life Technologies, Grand Island, NY, USA) at 42°C for 50 min and real-time polymerase chain reaction was performed. The following primers were used for gene amplification: µ-opioid receptor: sense GCCATCGGTCTGCCTGTAAT; antisense GAGCAGGTTCTCCCAGTAC, κ-opioid receptor: sense GTCAGAGGACAGCTTTGCAC, antisense TAGCTCAGTGAAGGTACATGC, δ-opioid receptor: sense ATGGTCATGGCAGTGACC, antisense CACGCAGATCTTGGTCACAG, Cyclofilin: sense 5-AATGCTGGACCAAACACAAA-3, antisense 5-CCTTCTTTCACCTTCCCAAA-3. The real-time PCR for the opioid receptors and house-keeper gene cyclofilin were run separately, and the amplification reactions were performed using the ABI Prism 7700 Sequence Detection System and the SYBR Green PCR Master Mix (Applied Biosystems Life Technologies, Grand Island, NY, USA). The results were quantified as Ct values, where Ct is defined as the threshold cycle of the polymerase chain reaction at which the amplified product is first detected. The expression levels were normalized using the cyclofilin expression level as an endogenous reference.

#### Immunoblot analysis

Frozen tissues were homogenized in cold RIPA buffer (1% Igepal CA-630, 0.5% sodium deoxycholate, 0.1% SDS, 1 mM PMSF, 10 mg/ml aprotinin, 1 mM sodium orthovanadate in PBS buffer, pH 7.4, 1∶300 protease and phosphatase inhibitor cocktail from Sigma-Aldrich, St. Louis, MO, USA (cat. number P8340, P5726, P0044)). The samples were centrifuged at 10,000× *g* for 10 min at 4°C. The supernatant was removed and centrifuged again. After centrifugation, the protein concentration in the supernatant was determined using Bradford assay [31]. Aliquots containing 80 µg of total protein were boiled in Laemmli loading buffer (BioRad, Hercules, CA, USA) and then loaded onto a 10% polyacrylamide gel. After separating by electrophoresis, the proteins were transferred to a nitrocellulose membrane (Bio-Rad, Hercules, CA, USA). The membranes were blocked in TBST (20 mM Tris-HCL, 150 mM NaCl, and 0.1% Tween 20) containing 5% non-fat dry milk for 2 h at room temperature and then incubated in µ-opioid receptor (C-terminus) rabbit IgG, δ-opioid receptor (C-terminus) rabbit IgG (1∶1000, Chemicon, Temecula, CA, USA, cat. numbers AB 5511, AB1560, respectively) or κ-opioid receptor-1 (N-19) goat polyclonal IgG antibody (1∶500; Santa Cruz Biotechnology, Dallas, TX, USA, cat. number sc31779) overnight at 4°C. The membranes were then incubated in the appropriate peroxidase-conjugated secondary antibody (1∶5000; anti-rabbit and anti-goat, Sigma-Aldrich, St. Louis, MO, USA, cat. numbers A0545 and A8919, respectively) for 90 min at room temperature and developed using enhanced chemiluminescence (Amersham GE Healthcare Bio-Sciences Corp.,; Piscataway, NJ, USA). The signal was detected by autoradiography. Quantification analysis of the blots was performed using the Scion Image software (Scion Corporation based on NIH image). Targeted bands were normalized to GAPDH (1∶5000, Advanced ImmunoChemical Incorporate, Long Beach, CA, USA).

### Opioid receptor activation studies: ELISA assay

#### *In vivo* studies

Naïve and PGE2-sensitized rats were injected with DAMGO (5 µg/paw), DPDPE (20 µg/paw), U 50,488 (10 µg/paw) or CRP (0.6 ng/paw). In the sensitized rats, these drugs were administered 2 h after the PGE2 injection. Approximately 1 h after the CRP or opioid receptor agonist treatment, the animals were perfused through the heart with phosphate-buffered saline and 4% paraformaldehyde (PFA) in 0.1 M phosphate buffer (PB, pH 7.4). The DRG (L5) and plantar tissue were removed, post-fixed for 4 h in 4% PFA, and transferred to a 30% sucrose solution in PB for 48 h to ensure cryoprotection. The tissues were sectioned (14 µm) on a cryostat, and the sections were mounted on histological glass slides covered with silane. Two sections from each region were circled using an ImmEdge PAP pen (Sigma-Aldrich) to form a water-proof barrier, and the resulting well could hold approximately 80 µl of solution. ELISA was performed in these wells using a 1∶500 dilution of primary antibody and a 1∶500 dilution of secondary antibody. To detect the activated receptor, we used antibodies that are able to distinguish the conformational change in the N-terminus of the opioid receptors following activation [32]. The antibodies were purchased from Proteimax Brazil. The reaction product was transferred to a 96-well plate, and the absorbance at 490 nm was measured using a microplate reader (Molecular Devices, Sunnyvale, CA, USA).2.4.3.2 Cell culture studies. These studies were performed using cultured DRGs obtained from naïve animals. Rats were decapitated, and all of the DRGs (45±50 DRGs) from the cervical, thoracic, lumbar and sacral levels were aseptically removed and collected in Earl's balanced salt solution (5 mM KC1, 116 mM NaCI, 1 mM NaH2PO4, 8 mM Na2HPO4, 1.46 mM KH2PO4, 8 mM NaHCO3 and 1% glucose). Next, the DRGs were digested in 0.5% collagenase (Sigma, Saint Louis, MO, USA) in EBSS at 37°C for 45 min. The tissue was then incubated for 15 min in EBSS containing 1% trypsin (Sigma, Saint Louis, MO, USA), washed in 2.5% trypsin inhibitor (Sigma, Saint Louis, MO, USA) and then triturated using a thin fire-polished pipette in Dulbecco modified Eagle medium (DMEM, Gibco Life Technologies, Grand Island, NY, USA) containing 1% HEPES buffer solution, penicillin/streptomycin (1∶200, Gibco, Life Technologies, Grand Island, NY, USA) and 10% heat inactivated fetal bovine serum (Sigma, Saint Louis, MO, USA). Next, the cells were subjected to density gradient centrifugation at 200×*g* for 1 min. The resulting pellet was resuspended in DMEM, and the cell suspension was filtered through a cell strainer (75 mm, Becton Dickinson Labware, Franklin Lakes, NJ, USA). The DRG cells were seeded onto 24-well culture plates at a density of 15,000 cells/well. The cells were cultured in a humidified incubator at 37°C with 5% CO2 and 95% air. Two days after seeding, the culture medium was changed. The cells were treated with PGE2 (1 µM) for 15 minutes for sensitization. Vehicle was used as a control. Cells were then treated with CRP dissolved in medium at three different concentrations (0.001, 1 and 100 µM) or DAMGO, DPDPE or U 50,488 (1 µM) for 15 minutes. In the yoked control wells, only culture medium was added (vehicle treatment). Three separate experiments were conducted on different occasions. For each experiment, new primary cultures were prepared under the same standard experimental conditions. Each treatment was replicated in three different wells. In the pilot studies, we determined the cell viability following the specific concentrations of agonists, CRP and PGE2 used. No significant increase in the number of trypan blue stained cells was observed after these treatments when compared with the vehicle (data not shown). ELISA was performed in each well as previously described.

#### Intracellular signaling studies

These studies were performed in DRG cell cultures obtained from naïve animals. The cells were obtained as previously described (section 2.3.3.2). CRP (1 µM) or U 50, 488 (1 µM) was added to the cells untreated or pretreated with PGE2 (1 µM) as previously described. In these studies, Nor-BNI (1 µM) was added to the culture (30 minutes before treatment). After treatment, the cyclic GMP content was measured using a cyclic GMP enzyme immunoassay kit purchased from Amersham Enzymeimmunoassay Biotrak (GE Healthcare Bio-Sciences Corp, Piscataway, NJ, USA).The phosphorylation of Akt, ERK 1/2 and JNK was evaluated by immunoblot analysis using the previously described method. The DRG cells were homogenized in a lysis buffer as previously described. The cells were incubated in primary antibody, either phosphorylated (p) AKT-T, pERK, pJNK, or non-phosphorylated forms of these proteins (1∶1000; Cell Signaling Technology, Danvers, MA, USA), overnight at 4°C and then incubated in HRP-conjugated secondary antibody (1∶2000; Amersham Biosciences, Arlington Heights, IL, USA). The blots were visualized using ECL solution for 1 min and exposed onto hyperfilms (Amersham Biosciences, Arlington Heights, IL, USA).

### Statistical analysis

The results are presented as the mean ± SEM. For figures 1, Fig. S1–4 the statistical evaluation of data was conducted using a two-way analysis of variance (ANOVA) with *post-hoc* testing by Tukey. One way ANOVA was used to analyze figures 2, 3, 4, 5, 6, 7, 8, 9, 10, with *post-hoc* testing by Tukey. A value of *P*<0.05 was considered significant.

**Figure 1:**
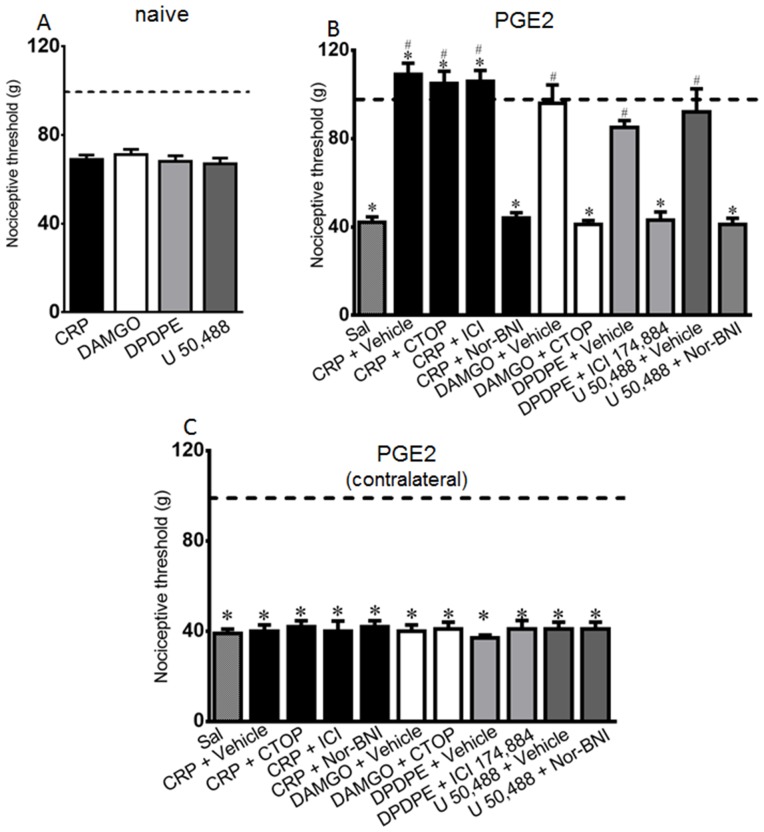
Intraplantar injection effect of crotalphine (CRP) or opioid receptor agonists in the rat nociceptive threshold. Pain threshold was obtained in the rat paw pressure test, before (dotted line) and 1 h after intraplantar injection of CRP (0.6 ng/paw), DAMGO (μ opioid receptor agonist, 5 µg/paw), DPDPE (δ opioid receptor agonist, 20 µg/paw), U-50488 (κ opioid receptor agonist, 10 µg/paw). CRP or opioid agonists were injected in naïve rats (Panel A) or in rats injected with PGE_2_ (100 ng/paw) 2 h before opioids or peptide administration (Panel B). CTOP (20 µg/paw), ICI 174,864 (ICI, 10 µg/paw) or nor-Binaltorphimine (NOR, 50 µg/paw), μ, δ and κ opioid receptor antagonists, respectively, or vehicle (50 µl/paw) were injected by the intraplantar route immediately before the opioid agonists or CRP administration. Panel C represents PGE_2_-induced hyperalgesia in the contralateral paw. Data represent mean values ± S.E.M. for six rats per group. * significantly different from baseline (dotted line), # significantly different from control (saline = SAL). Data were analyzed by two-way analysis of variance (ANOVA) with *post-hoc* testing by Tukey.

**Figure 2:**
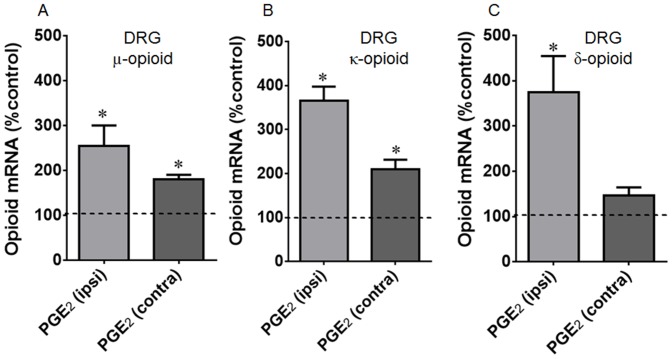
Intraplantar injection effect of prostaglandin E_2_ (PGE_2_) on gene (A, C, E) expression of μ (A), κ (B) and δ (C) opioid receptors. The changes in mRNA levels of opioid receptors were determined by real time PCR, in dorsal root ganglia (DRG) obtained 3 h after PGE_2_ injection (100 ng/paw). Dotted line represents the values obtained in naïve animals. Data are presented as mean of 7 animals ± SEM and expressed as % of control (naïve) animals. * Significantly different from mean values of naïve animals (p<0.05). Data were analyzed by one-way analysis of variance (ANOVA) with *post-hoc* testing by Tukey.

**Figure 3:**
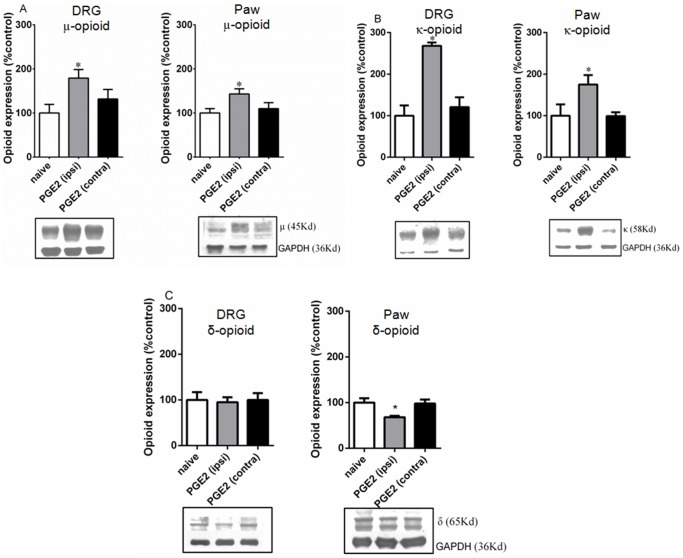
Intraplantar injection effect of prostaglandin E_2_ (PGE_2_) on protein expression of μ (A), κ (B) and δ (C) opioid receptors. The changes in protein levels of opioid receptors were determined by immunobloting, in dorsal root ganglia (DRG) and plantar tissue obtained 3 h after PGE_2_ injection (100 ng/paw). Data are presented as mean ± SEM and expressed as % of control (naïve) animals. *Significantly different from mean values of naïve animals, n = 6 per group (p<0.05). Data were analyzed by one-way analysis of variance (ANOVA) with *post-hoc* testing by Tukey.

**Figure 4:**
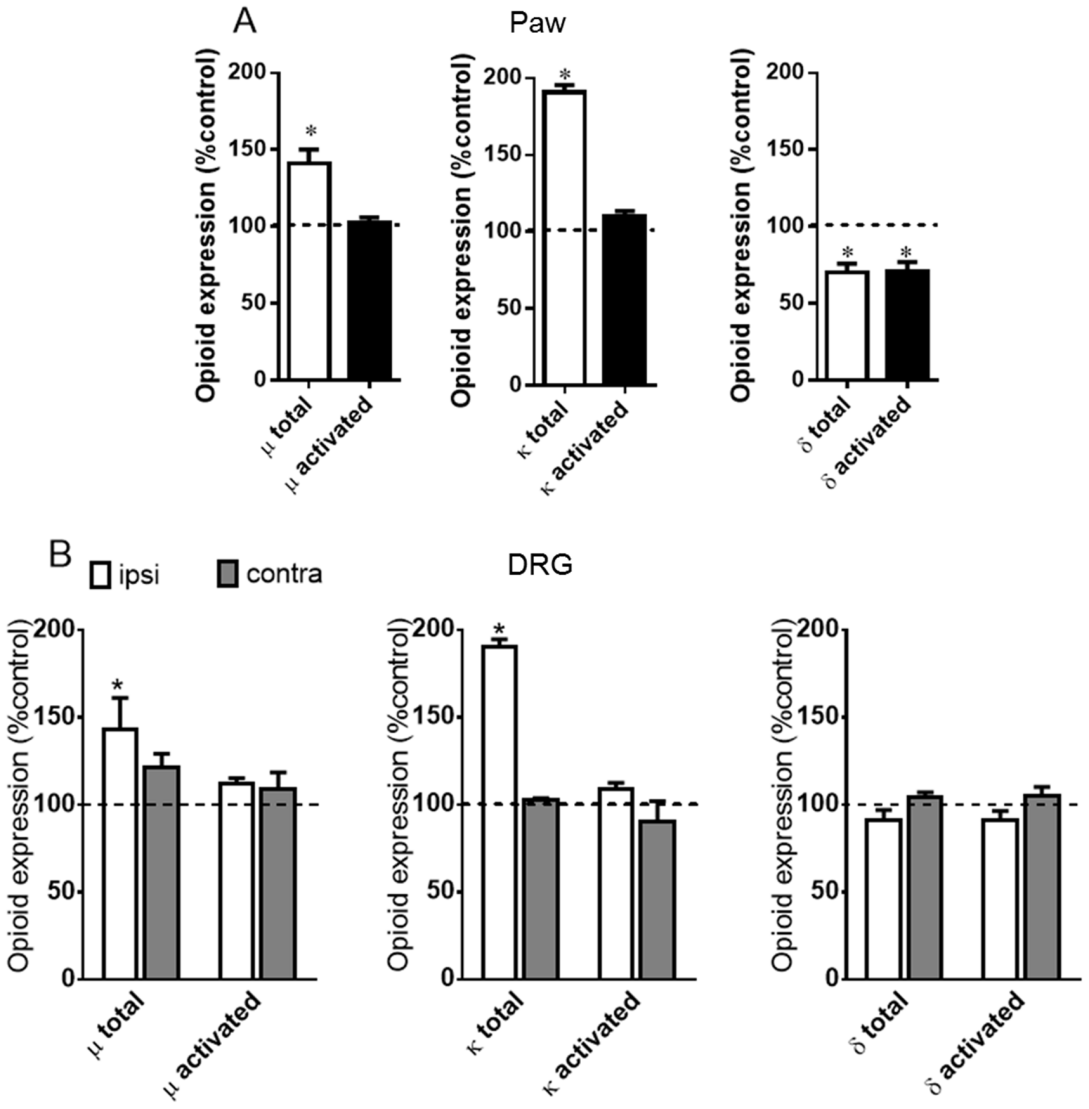
PGE2, *per se*, does not activate opioid receptors. The plantar tissue (**A**) and dorsal root ganglia (ipsi and contralateral) (**B**) were obtained 3 h after PGE2 injection (100 ng/paw). Tissues obtained from naïve animals were used as controls. The tissues were sectioned and probed with anti-μ, κ or δ antibodies. (**A**) The black bar represents the results obtained with the conformation state-sensitive antibodies whereas the white bar represents the results obtained using the control antibodies (western blotting assay antibodies). (**B**) The first couple of bars represents the results obtained using the control antibodies (western blotting assay antibodies). The second one represents the conformation state-sensitive antibodies. The dotted line represents the values obtained for naïve animals. * Significantly different from mean values of naïve animals (p<0.05). Data were analyzed by one-way analysis of variance (ANOVA) with *post-hoc* testing by Tukey.

**Figure 5:**
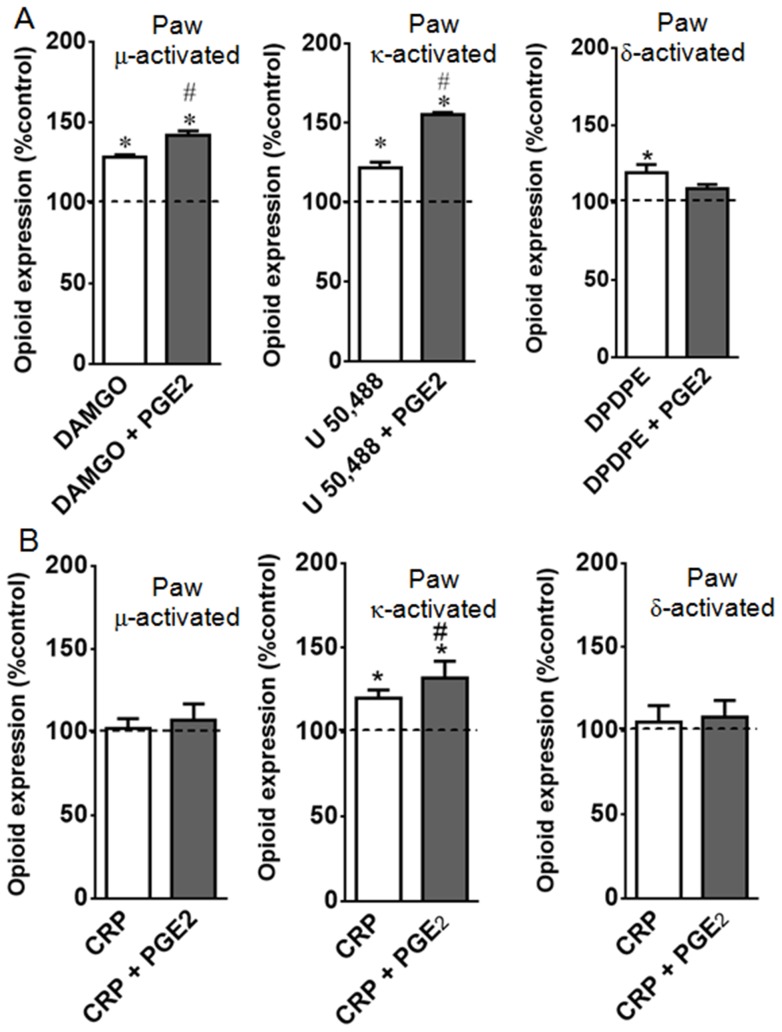
PGE_2_ improves agonist-mediated opioid receptor activation. Naïve or prostaglandin E2 (PGE_2_, 100 ng/paw) injected rats were treated with DAMGO (5 µg/paw), U50,488 (10 µg/paw), DPDPE (20 µg/paw), (Panel A) or crotalphine (CRP, 0.6 ng/paw) (Panel B). The agonists, CRP, or vehicle (control) were administered 2 h after PGE2 injection. ELISA assay was performed in slices of plantar tissue, using conformation state-sensitive antibodies (μ, κ, δ activated). Tissues were collected 3 h after PGE_2_ injection. *Significantly different from mean values of control animals (dotted line), n = 6 animals per group (p<0.05). #Significantly different from mean values of CRP or agonist alone (p<0.05). Data were analyzed by one-way analysis of variance (ANOVA) with *post-hoc* testing by Tukey.

**Figure 6:**
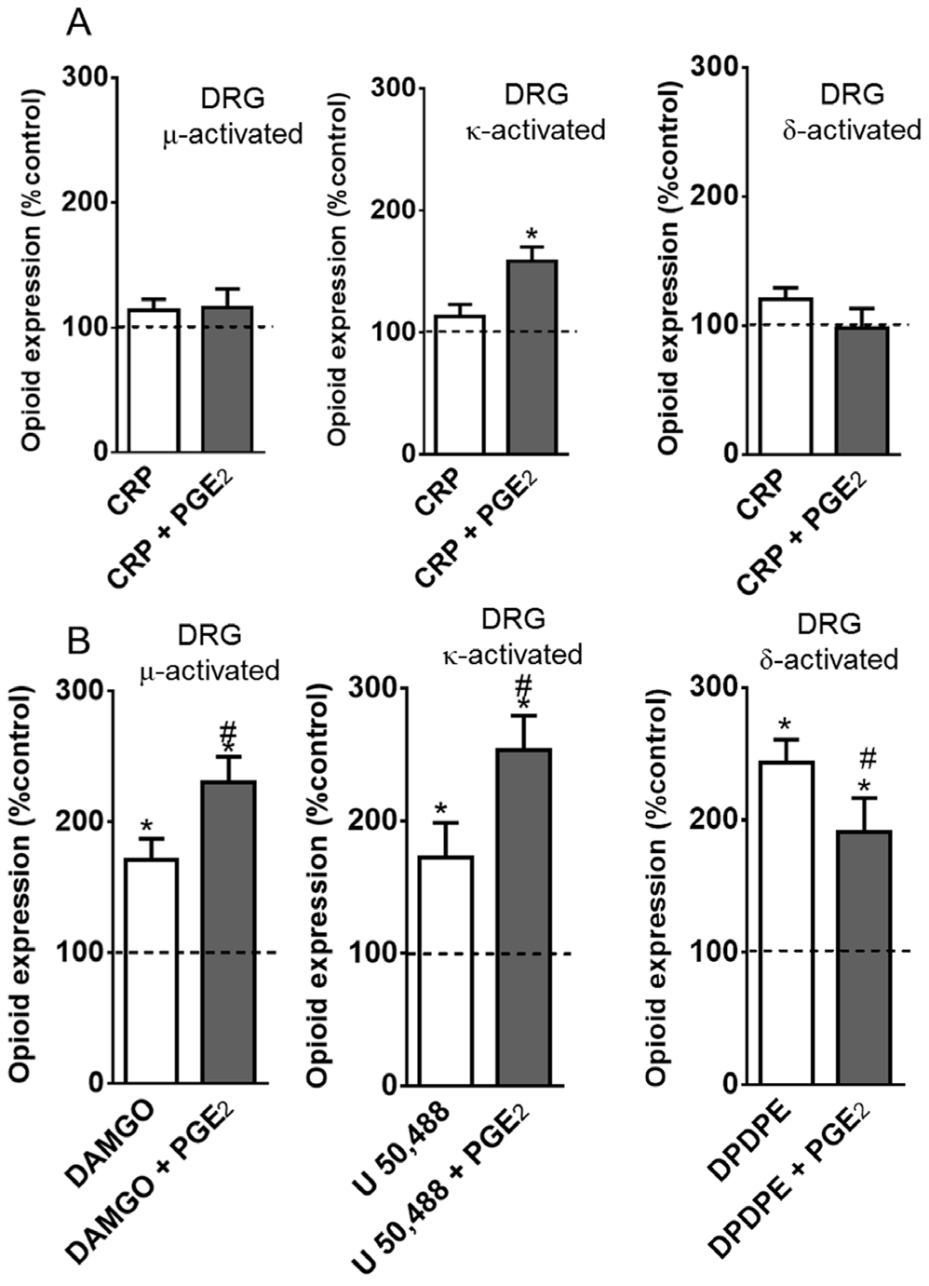
PGE_2_ improves agonist-mediated opioid receptor activation in primary neuronal cell culture. Cells from dorsal root ganglia were obtained from naïve rats and incubated at 37°C, in a 5% of CO_2_, for two days. On the third day, the cells were incubated with PGE2 (1 µM) or vehicle for 15 min and further incubated with 1 µM of crotalphine (CRP, A) or DAMGO, U50,488, DPDPE (B) for 15 min. ELISA assay was performed directly on fixed cells, using conformation state-sensitive antibodies (activated). Three separate experiments were carried out on different occasions.* Significantly different from mean values of naïve animals (dotted line) (p<0.05), # Significantly different from mean values of CRP or agonist alone, average of 3 independent experiments (p<0.05). Data were analyzed by one-way analysis of variance (ANOVA) with *post-hoc* testing by Tukey.

**Figure 7:**
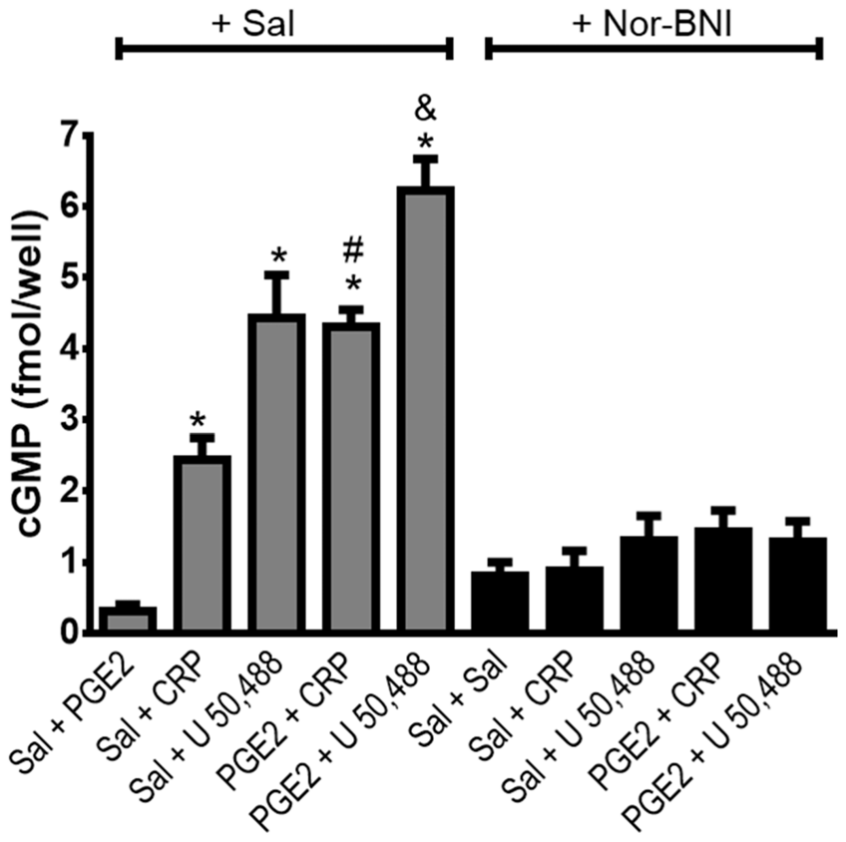
Effect of crotalphine on cyclic GMP levels in primary culture of DRG cells. Cells from DRG were obtained from naïve rats and incubated at 37°C in a 5% of CO_2_, for two days. On the third day, the cells were incubated with Nor-BNI (1 µM) for 30 min and PGE_2_ (1 µM) or vehicle (saline - Sal) for 15 min, and further incubated with 1 µM of crotalphine (CRP), U50,488, or saline, for 15 min. Cells were lysed and subjected to enzyme immunoassay.*Significantly different from mean values of control cells (p<0.05), # Significantly different from mean values of CRP alone (p<0.05), Significantly different from mean values of U 50,488 alone (p<0.05). cGMP levels were analysed by one-way analysis of variance with a post-hoc Tukey test. Data were analyzed by one-way analysis of variance (ANOVA) with *post-hoc* testing by Tukey.

**Figure 8:**
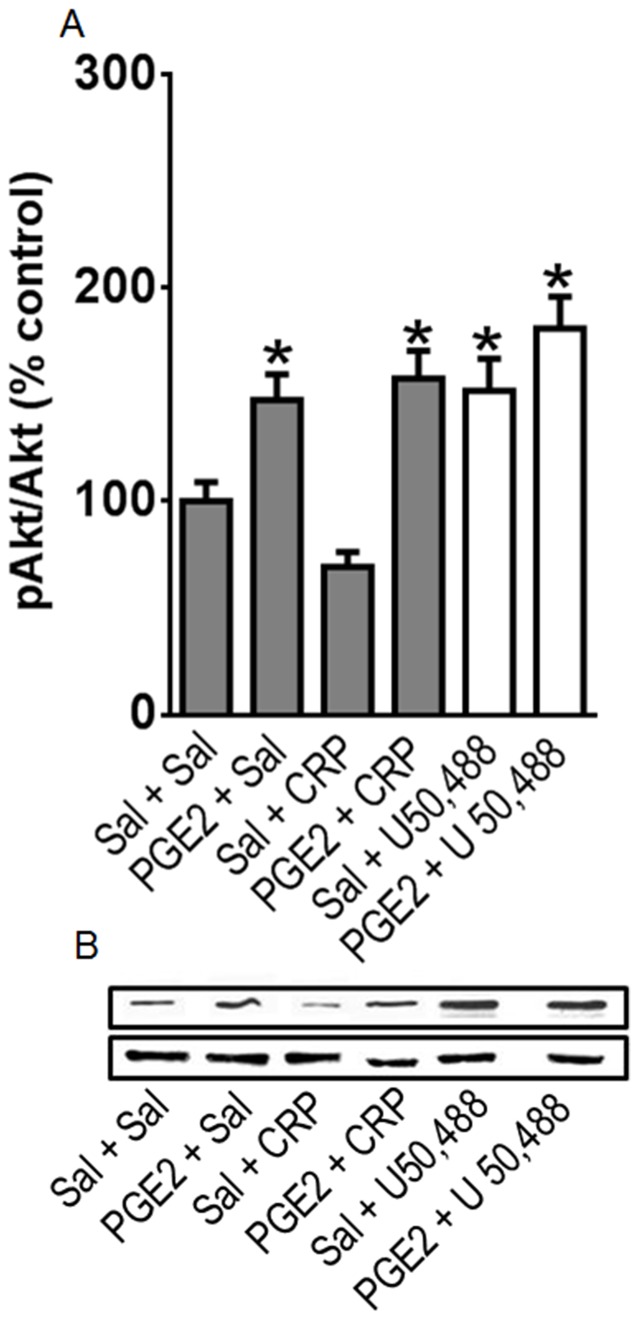
Effect of crotalphine (CRP) on AKT phosphorylation in primary culture of DRG cells. Cells from DRG were obtained from naïve rats and incubated at 37°C in a 5% of CO_2_, for two days. On the third day, the cells were incubated with PGE_2_ (1 µM) or vehicle (saline - Sal) for 15 min. and further incubated with 1 µM of CRP, U50,488, or saline, for 15 min. Cells were scrapped and homogenized, and the total homogenate was subjected to western blotting. (A) The ratio of phospho-protein to total protein is expressed as percentage from control (Sal+Sal). Three separate experiments were carried out on different occasions.*Significantly different from mean values of control cells (p<0.05). AKT levels were analysed by one-way analysis of variance with a *post-hoc* Tukey test. (B) Representative blots showing the levels AKT in the total lisate of DRGs.

**Figure 9:**
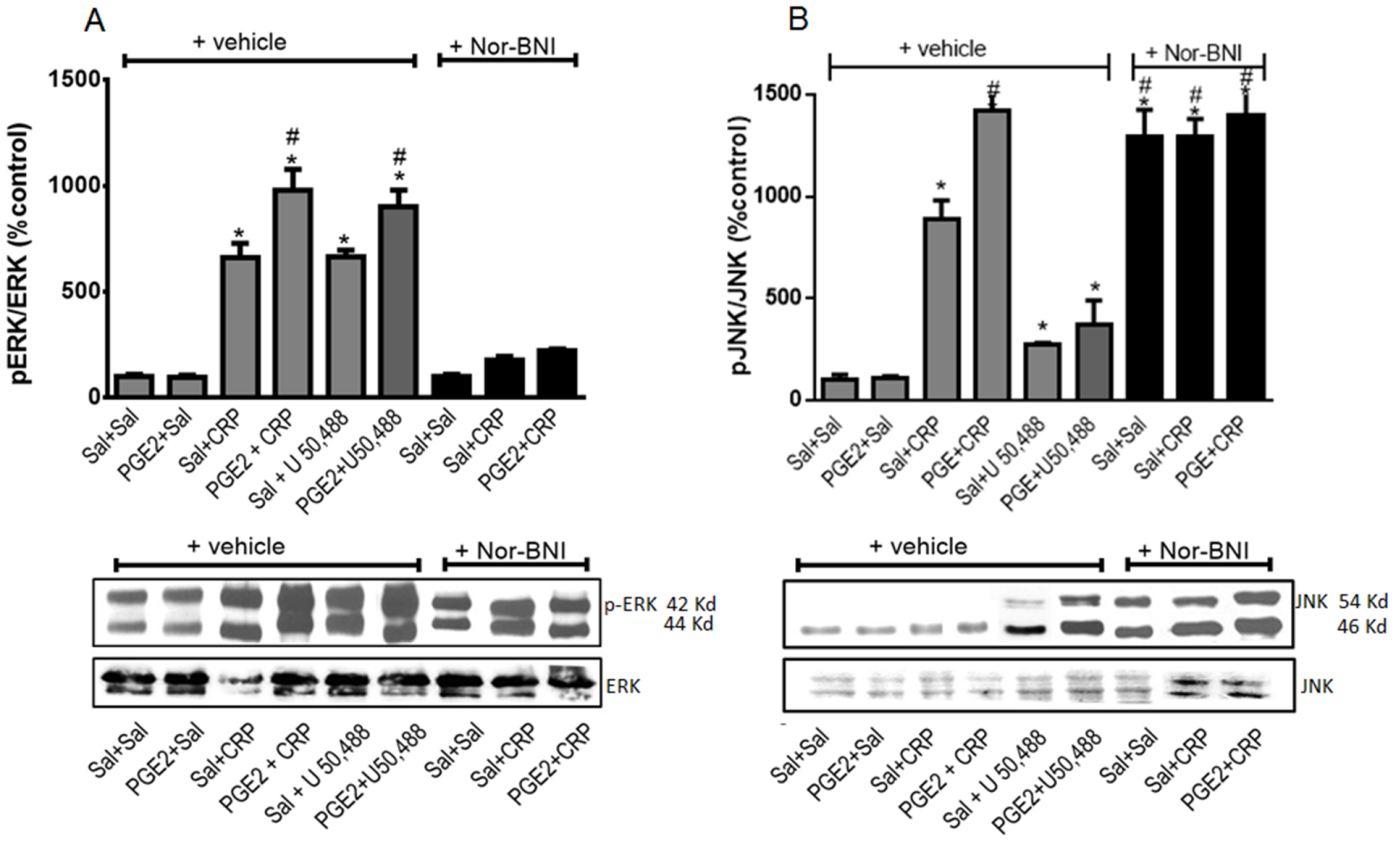
Effect of crotalphine (CRP) on phosphorylated form of ERK (A) and JNK (B) in primary culture of DRG cells. Cells from DRG were obtained from naïve rats and incubated at 37°C in a 5% of CO_2_, for two days. On the third day, cells were incubated with Nor-BNI (1 µM) for 30 min and PGE_2_ (1 µM) or vehicle (saline - Sal) for 15 min and further incubated with 1 µM of CRP, U50,488, or saline, for 15 min. Cells were scrapped and homogenized, and the total homogenate was subjected to western blotting. The ratio of phospho-protein to total protein is expressed as % of control (sal+sal). GAPDH was used as the internal loading control. Three separate experiments were carried out on different occasions.*Significantly different from mean values of control cells (p<0.05). (A) p-ERK1/2 and (B) p-JNK levels were analysed by one-way analysis of variance with a *post-hoc* Tukey test. Representative blots showing the levels p-ERK1/2 and p-JNK in the total lysate of DRGs. # Significantly different from mean values of CRP alone (p<0.05).

**Figure 10:**
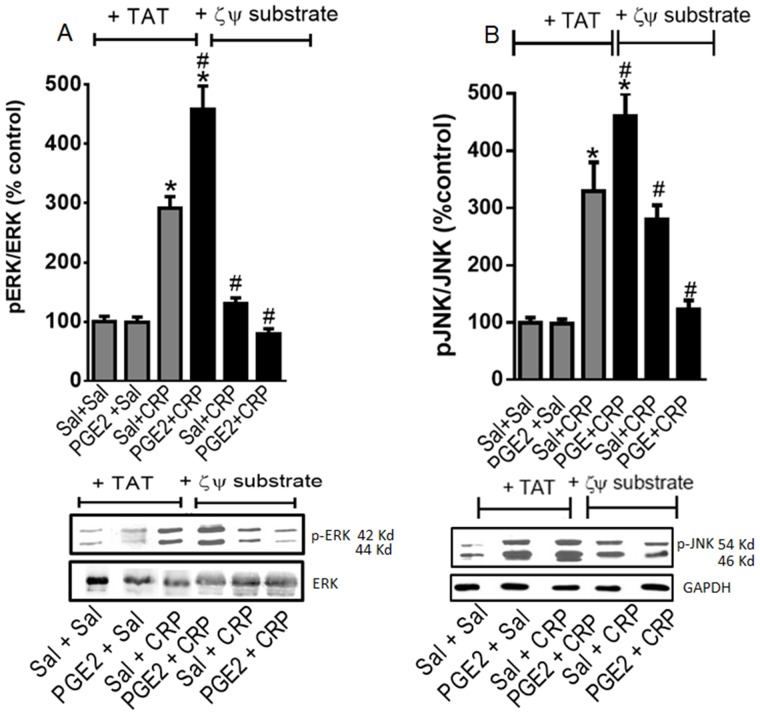
Involvement of PKCζ on crotalphine-mediated ERK1/2 and JNK phosphorylation in DRG primary culture. Cells from DRG were obtained from naïve rats and incubated at 37°C in a 5% of CO_2_, for two days. On the third day, cells were incubated for 30 minutes with TAT carrier (control) or ζ pseudosubstrate (1 µM) followed by incubation with PGE_2_ or vehicle (15 minutes). The neurons were then incubated with 1 µM of crotalphine (CRP) and TAT or ζ pseudosubstrate for 15 minutes. Cells were scrapped and homogenized, and the total homogenate was subjected to western blotting for (**A**) pERK1/2 and (**B**) pJNK. The ratio of phospho-protein to total protein isexpressed as % of control (Sal+Sal). GAPDH was used as the internal loading control. Three separate experiments were carried out on different occasions.*Significantly different from mean values of control cells (p<0.05). # Significantly different from mean values of CRP alone (p<0.05). p-ERK1/2 and p-JNK levels were analysed by one-way analysis of variance with a *post-hoc* Tukey test. Representative blots showing the levels pERK1/2 or pJNK in the total lysate of DRGs.

## Results

### Acute sensitization improves the anti-nociceptive effect of CRP and opioid receptor agonists

The intraplantar injection of CRP (0.6 ng/paw) or the opioid agonists DAMGO (5 µg/paw), U-50488 (10 µg/paw) or DPDPE (20 µg/paw), which were used as positive controls, did not modify the basal nociceptive threshold of naïve rats in the rat paw pressure test (Fig. 1A). The intraplantar injection of PGE2 caused a significant decrease in the nociceptive threshold, with a peak response occurring 3 h after the administration (Fig. 1B, checkered bar, and Fig. S1), compared with the basal values obtained before any treatment, which is a characteristic sign of hyperalgesia. The administration of CRP or opioid receptor agonists, in the same doses previously used [11], [25], blocked the PGE2-induced hyperalgesia and increased the nociceptive threshold of the animals when compared with the basal values (Fig. 1B). These effects were only observed in the paw injected with CRP or agonists. These treatments did not modify the PGE2-induced hyperalgesia in the contralateral paw (Fig. 1C). In order to confirm the peripheral CRP and opioid agonists effect, rats were treated with naloxone methiodide, a peripherally acting opioid antagonist. This antagonist blocked CRP and opioid agonists-induced anti-nociception (Fig. S2). In addition, the CRP or opioid receptor agonists systemic injection, in the same doses injected intraplantarly, did not alter PGE2-induced hyperalgesia (Fig. S3). Together, these results indicate that CRP and the opioid receptor agonists, in the doses used in this study, have a local anti-nociceptive effect in the presence of the acute sensitization induced by PGE2.

Previously, we demonstrated that the anti-nociceptive effect of CRP administered orally in this hyperalgesia model is mediated by the activation of the peripheral (local) κ-opioid receptor [11]. To determine whether opioid receptors are involved in the local (intraplantar) anti-nociceptive effect of this peptide, rats were administered selective antagonists of the opioid receptors by i.pl. injection. The anti-nociceptive effect of CRP was abolished in the paw injected with nor-BNI, an antagonist of the κ-opioid receptors (Fig. 1B), but not by CTOP or ICI 174,864, μ- and δ-opioid receptor antagonists, respectively (Fig. 1B). These results indicate that the peripheral anti-nociceptive effect of CRP is mediated by the activation of the peripheral κ-opioid receptor. The opioid receptor antagonists CTOP, ICI 174,864, and Nor-BNI blocked the anti-nociceptive effects induced by their corresponding agonist (DAMGO, DPDPE, and U 50,488, respectively), which confirms the efficacy of the antagonist doses used (Fig. 1B). The antagonists, *per se*, had no effect on the hyperalgesia induced by PGE2 (data not shown).

### Acute sensitization interferes with the opioid receptor levels

To elucidate the potential mechanisms involved in the anti-nociceptive effect of CRP and the opioid agonists observed in the presence of prostaglandin E2 sensitization, we investigated whether this eicosanoid treatment alters the mRNA expression levels of the opioid receptors. PGE2 treatment significantly increased the mRNA expression levels of the µ- and κ-opioid receptors in the ipsilateral (2.5- and 3.6-fold, respectively) and contralateral (0.8- and 2-fold, respectively) DRGs compared with the DRGs obtained from naïve animals (Figs. 2A, 2B). An increase in the mRNA expression level of the δ opioid receptor was only detected in the ipsilateral DRGs (3.7-fold) (Fig. 2C).

To determine whether the changes in the mRNA levels resulted in changes in the opioid receptor protein expression levels, we performed immunoblot assays using the DRGs and plantar tissue. The intraplantar injection of PGE2 increased the µ- and κ-opioid receptor protein expression levels in the ipsilateral DRGs (79% and 168%, respectively) and the plantar tissue (43% and 75%, respectively) compared with the tissues obtained from naïve animals (Figs 3A and B). In contrast, a 30% decrease in the δ-opioid receptor protein expression level was detected in the plantar tissue from the PGE2 injected animals (Fig. 3C). Despite the changes detected in the ipsilateral DRGs mRNA levels, no PGE2-related changes in the µ-, κ-, and δ-opioid receptor protein expression levels were detected in the contralateral DRGs (Fig. 3). PGE2-induced hyperalgesia was confirmed in all animals used for mRNA and immunoblot (Fig. S4).

### PGE2 sensitization enhances the CRP and agonist-mediated opioid receptor activation

It has been well established that GPCRs undergo conformational changes in the N-terminus following receptor activation [32]. To determine whether PGE2 activates opioid receptors, we performed an ELISA assay (in plantar tissue and DRG slices) using conformation state-sensitive antibodies that recognize activated opioid receptors according to the methods published by Gupta et al. (2007). To determine the total expression level of the opioid receptors, the same antibodies used for the immunoblot assays were also used in these assays.

Consistent with the immunoblot assay results, the intraplantar injection of PGE2 increased the expression levels of the µ- and κ-opioid receptors in the plantar tissue (43% and 72%, respectively) (Fig. 4A) and the DRG (43% and 97%, respectively) (Fig. 4B) and decreased the expression level of the δ opioid receptor in the plantar tissue (30%). No changes in opioid receptor activation, however, were detected using the conformation state-sensitive antibodies compared with the levels observed in the tissues from naïve animals. This result indicates that PGE2 does not activate opioid receptors.

Because PGE2 altered the expression profiles of the opioid receptors without altering their activation, we investigated whether CRP or opioid receptor agonists increase the activation of the opioid receptors in PGE2-sensitized plantar tissue. The results show that the intraplantar injection of DAMGO (5 µg/paw), U 50,488 (10 µg/paw) and DPDPE (20 µg/paw) into naïve animals results in conformational changes in the µ-, κ- or δ-opioid receptors, respectively, when compared with the untreated rats (Fig. 5A). Interestingly, CRP (0.6 ng/paw) caused κ- but not µ- or δ-opioid receptor activation (Fig. 5B). These results indicate that these opioid receptors have been activated and confirm the involvement of the κ-opioid receptor in the CRP-mediated anti-nociceptive effect. The intraplantar injection of PGE2 increases the opioid receptor activation caused by CRP and the µ- and κ-opioid receptor agonists (32%, 42% and 36%, respectively) compared with the values obtained without sensitization (Figs. 5A, 5B). In contrast, PGE2 inhibited the activation of the δ- opioid receptor compared with the values obtained from the tissues collected from the animals that were only treated with the δ-opioid agonist. Our results suggest that acute sensitization increases the activation of the opioid receptors by CRP and, depending on the type of opioid receptor, by selective agonists.

To confirm our *in vivo* findings, the effect of CRP and opioid receptor agonists on opioid receptor activation was evaluated using primary neuron cultures isolated from the DRGs of adult naïve rats. CRP added to the cell culture medium did not result in conformational changes in the µ-, δ- or κ-opioid receptors compared with the naïvecontrols (Fig. 6A). Conversely, treating PGE2-sensitized DRG cells with CRP this peptide now induces conformational changes in the κ-opioid receptor but not in the µ- or δ-opioid receptors when compared with the cells treated with CRP but not PGE2 (Fig. 6A). These results indicate that PGE2triggers the CRP activation of the κ-opioid receptor. Incubating the DRG cells in the opioid receptor agonists DAMGO, U 50,488 and DPDPE resulted in the activation of their corresponding µ-, κ -, and δ-opioid receptors (70%, 72% and 143%, respectively, Fig. 6B). The activation of the µ- and κ-opioid receptors increased significantly when the cells were pretreated with PGE2. Interestingly, pretreating the DRG neurons with PGE2 inhibited the DPDPE activation of the δ-opioid receptor compared with the cells treated with the agonist alone (Fig. 6B).

### PGE2 sensitization enhances the CRP-mediated κ-opioid receptor intracellular signaling

#### Effect on cyclic GMP level

Because the anti-nociceptive effect of CRP is mediated by the L-arginine-nitric oxide-cGMP pathway [12], we investigated the effect of CRP on the cyclic GMP (cGMP) level in DRG cell cultures. CRP (1 µM) increased the cGMP level compared with the control cells (Fig. 7), and pretreating the cells with PGE2 (1 µM) significantly increased (30%) the CRP-induced cGMP release. Nor-BNI, a κ-opioid receptor antagonist, blocked this effect. The κ-opioid receptor agonist U 50,488 (1 µM) was used as a positive control and increased the cGMP level. Pretreating the cells with PGE2 further increased (33%) the cyclic nucleotide level compared with the cells that were treated with only the agonist (Fig. 7).

#### Effect on AKT activation

AKT phosphorylation is involved in the peripheral anti-nociceptive effect of opioids, such as morphine and U 50,488 [18], [33]. Therefore, we evaluated whether CRP increases AKT phosphorylation in PGE2-sensitized cells.

Treating DRG cells with PGE2 (1 µM) increased the AKT phosphorylation level compared with the saline-treated (control) cells (Fig. 8), and CRP (1 µM) did not interfere with AKT phosphorylation compared with the control cells and the PGE2-treated cells (Fig. 8). The κ-opioid receptor agonist U 50,488 (1 µM) was used as a positive control and activated AKT (Fig. 8).

#### Effect on MAPK activation

The activation of the κ-opioid receptor increases the phosphorylation of MAPKs (ERK1/2 and JNK) in neuronal and non-neuronal cells [34]. Therefore, MAPK phosphorylation may be a useful indicator of opioid receptor activation in DRG cells. CRP increased ERK1/2 phosphorylation in DRG cells (Fig. 9A), and pretreating the cells with PGE2 significantly increased (1.5-fold) the CRP-mediated ERK1/2 phosphorylation (Fig. 9A). PGE2 alone, however, does not appear to induce ERK1/2 phosphorylation. The κ-opioid receptor antagonist Nor-BNI inhibited the CRP-mediated ERK1/2 phosphorylation, which suggests that the κ-opioid receptor is involved in this process. Nor-BNI alone does not appear to cause ERK1/2 phosphorylation. Based on these results, we further evaluated the effect of the κ-opioid receptor agonist U 50,488 on ERK1/2 activation. Treating neurons with U 50,488 increased the level of phosphorylated ERK1/2, and the phosphorylated ERK1/2 level was further increased in cells that were pretreated with PGE2 (Fig. 9A). Treating neurons with either CRP or U 50, 488 also induced JNK phosphorylation (9- and 2,5-fold, respectively). Similar to the ERK1/2 expression profile, pretreating the cells with PGE2 increased the CRP- and U 50, 488-mediated JNK phosphorylation (10.5- and 3.5-fold, respectively). PGE2 alone does not appear to induce JNK phosphorylation. Nor-BNI did not interfere with this CRP effect, but this antagonist did increase the level of phosphorylated JNK (13-fold) (Fig. 9B).

#### PKC ζ is involved in CRP-induced MAPK activation

Because CRP appears to activate ERK and JNK, we further investigated the potential mechanisms involved in this effect. Previous studies indicate that PKCζ is involved in the activation of ERK1/2 by the κ-opioid receptor agonist [20]. To examine the direct role that PKC-ζ plays in the activation of the MAPKs by CRP, we used the PKC-ζ pseudosubstrate, which selectively inhibits the atypical PKC ζ isozyme [35]. Pretreating the DRG cells with the PKC ζ pseudosubstrate abolished the CRP-mediated ERK1/2 phosphorylation in both the PGE2-sensitized and unsensitized cells (Fig. 10A). In contrast, the ζ pseudosubstrate did not affect the CRP-mediated JNK phosphorylation but did inhibit the increase in CRP-induced JNK phosphorylation observed in the presence of PGE2 (Fig. 10B). The TAT carrier was used as a negative control.

## Discussion

We previously reported that CRP, a 14-aminoacid peptide isolated from C. d. terrificus venom, has a potent, long-lasting and peripheral opioid receptor-mediated anti-nociceptive effect [11]–[13]. In this study, we demonstrated that the potency and long-lasting anti-nociceptive effect of CRP depends on tissue inflammation. It has been well established that the local efficacy of opioid drugs is enhanced in the presence of tissue injury/inflammation, but the mechanisms involved in this phenomenon remain under investigation [6], [7], [9]. In the present study, we also elucidated some of the mechanisms involved in the increased peripheral anti-nociceptive efficacy of CRP and opioid receptor agonists in the presence of hyperalgesia. We observed the following results: (a) the local intraplantar (i.pl.) injection of a low dose of CRP or selective opioid receptor agonists, which are ineffective at increasing the nociceptive threshold in naïve animals in the rat paw pressure testnaïve, produced anti-nociception in a PGE2-induced hyperalgesia modelPGE2. In this hyperalgesia rat model, the CRP effect was mediated by activation of the κ-opioid receptor. (b) The i.pl. administration of PGE2 increased the peripheral mRNA expression levels of the µ-, δ-, and κ-opioid receptors and the peripheral protein expression levels of the µ- and κ-opioid receptors within 3 h of the injection. The eicosanoid also increased the activation of the µ- and κ-opioid receptors by their corresponding agonist. In contrast, the PGE2 treatment decreased the δ-opioid receptor protein level and activation of the δ-opioid receptor by its agonist. (c) In cultured DRG neurons, the activation of specific intracellular signaling pathways (MAPKs and PKCζ) by CRP was enhanced following PGE2 sensitization. The results presented here provide evidence that tissue injury regulates the expression of peripheral opioid receptors and their activation by CRP and selective opioid receptor agonists and that tissue injury also contribute to activation of specific intracellular signaling pathways.

In our study, we first evaluated the effect of an intraplantar injection of CRP and selective agonists of the µ-, κ- and δ-opioid receptors on the nociceptive threshold of animals that were sensitized or not by an i.pl. injection of PGE2. This eicosanoid sensitizes the peripheral terminals of the neuron, which reduces the threshold for activating the nociceptors [36] [37] [38]. The i.pl. injection of CRP and DAMGO, DPDPE and U 50,488 (µ-, δ- and κ-opioid receptor agonist, respectively), in doses that are ineffective at altering the mechanical nociceptive stimuli withdrawal response in naïve rats, blocked PGE2-induced hyperalgesia. Furthermore, we should mention that the doses of the agonists and antagonists used in this study were chosen based on their optimal effects as observed in previous dose-response studies [13], [23]. The doses used in the present study only had a local effect because anti-nociception was only detected in the paw injected with the drugs and not in the contralateral paw. Moreover, the agonists and CRP effects were reversed by naloxone methiodide, a peripherally acting opioid antagonist. Additionally, the drug's effect seems to be restricted to the receptors in the injury site, because a systemic subcutaneous injection of the equivalent dose of drugs used intraplantarly did not induced anti-nociception. We also demonstrate that the effect of CRP was inhibited by the i.pl. injection of the κ-opioid receptor antagonist but that it was unaffected by the injection of the µ- or δ-opioid receptor antagonists. These results are consistent with our previous results showing that the effect of CRP administered orally is mediated by the κ-opioid receptor in acute pain models [11]. The efficacy of the opioid receptor antagonist doses used was confirmed by the results showing that the i.pl. injection of the antagonists blocked the peripheral anti-nociceptive effect of their corresponding agonist.

Taken together, these results provide evidence that the local anti-nociceptive effects of CRP and opioids depend on previous sensitization. These findings are consistent with the clinical and experimental observations reporting that opioids are more potent under inflammatory conditions [4], [5], [39]–[42]. The mechanisms involved in this phenomenon have not been fully elucidated, but biochemical assay studies have shown that inflammation/tissue injury enhances the axonal transport of opioid receptors toward the periphery, which is preceded by an increase in the opioid receptor mRNA transcription level [3], [10]. Importantly, peripheral opioid receptor levels do not increase during the early stages of the inflammatory process, but these levels do increase at later stages of the inflammation [43], [44]. Based on these findings and the lack of experimental data showing the effect of PGE2 on the opioid receptor expression levels, we next investigated whether this eicosanoid affects the expression levels of the opioid receptors. Our results indicate that PGE2 sensitization increases the mRNA and protein expression levels of the µ- and κ-opioid receptors in the dorsal root ganglia and plantar tissue of rats. Interestingly, PGE2 appears to increase the δ-opioid receptor mRNA expression level in the DRGs while simultaneously PGE2 decreasing the δ-opioid receptor protein level in the paw. This discrepancy may be a consequence of protein degradation during axonal transport. Further studies, however, are needed to investigate these differences. Furthermore, despite an increase in the δ-opioid mRNA expression level in the DRGs, the protein level remained unchanged in this tissue. An evaluation of the protein expression level at later time points may explain whether this discrepancy is a time point issue. Studies have shown that inflammatory substances, such as Freund's complete adjuvant (FCA) and carrageenan, increase the mRNA and protein expression levels of the µ-opioid receptor in the DRGs within 1 h after treatment [3], [6]. These discrepancies suggest that µ- and δ-opioid receptor require different timing to be up or down regulated during inflammation. We further examined whether PGE2 induces opioid receptor activation by performing ELISA using plantar tissue slice preparation and conformation state-sensitive antibodies specific to activated opioid receptors (the N-terminal region of GRPCs undergo a conformational change following receptor activation that can be selectively detected by these specific antibodies [32], [45], [46].). Our results show that PGE2 did not alter the activation state of these receptors, though the opioid receptor expression levels increased.

Next, we determined whether sensitization by this eicosanoid improves the agonist-mediated receptor activation. Using the state-sensitive antibodies we detected a higher level of µ- and κ-opioid receptor activation by their corresponding agonists in PGE2-sensitized cells.

Pretreating the cells with PGE2 inhibited δ-opioid receptor activation by its agonist. This result could be the consequence of the PGE2-mediated δ-opioid receptor down-regulation. Zollner et al. (2003) showed that despite increasing the number of µ-opioid receptor binding sites on the DRG cell membrane, the intraplantar injection of FCA does not affect the affinity of the full and partial µ-opioid agonists in rats [7]. Furthermore, one important finding from their study is that there was a significant increase in the DAMGO-stimulated G-protein activation in FCA-treated animals.

The results from the present study demonstrate that CRP treatment is sufficient for activation of the κ-opioid receptor in the presence of PGE2. CRP, however, may not directly activate opioid receptors [11]. Another study showed that the anti-nociceptive effect of CRP can be reversed by dynorphin A anti-serum, which indicates that endogenous opioids are involved in this phenomenon (Machado et al., in press). We still cannot dismiss, however, that CRP may have a direct effect on opioid receptors. Nevertheless, the involvement of an opioid mechanism in the CRP anti-nociceptive effect is supported by the results obtained in this study and in previous studies [11]–[13].

Based on our *in vivo* findings, we next wanted to determine the effect of CRP on opioid receptor activation in PGE2-sensitized DRG cells isolated from naïve animals. PGE2 increased the CRP-mediated κ-opioid receptor activation. This result is consistent with our *in vivo* result showing that the CRP anti-nociceptive effect in the PGE2-induced hyperalgesia model is mediated by κ- opioid receptor activation.

Because inflammation increases the frequency of interactions between opioid receptors and G proteins and activates specific intracellular signaling pathways [7], [47], we decided to investigate the molecular pathways activated by CRP in the presence or absence of PGE2 sensitization. It has been shown that the opioid receptor-mediated anti-nociceptive effect is regulated by the cGMP, AKT, and MAPK signaling pathways [20], [48]–[50]. Furthermore, we recently showed that cGMP is involved in the CRP anti-nociceptive effect [12]. In the present study, we demonstrated that CRP increases the cGMP level and that this effect is enhanced by PGE2. AKT activation is also involved in the peripheral anti-nociception induced by opioids, such as morphine and U 50,488 [18], [33]. Our cell culture results indicate that the κ-opioid receptor agonist (U 50,488), used as a positive control, and not CRP, phosphorylates AKT. Interestingly, recent findings obtained by our group indicate that the PI3-AKT signaling pathway is not involved in the CRP anti-nociceptive effect in the PGE2-induced hyperalgesia model (Pedroso and Cury, unpublished data). CRP and the κ-opioid receptor agonist activated ERK1/2 and JNK. It is important to mention that in all of the experiments, PGE2 increased the phosphorylation levels of MAPK caused by CRP. The role that MAPKs play in the CRP-mediated anti-nociceptive effect is currently unknown. It is possible that the activation of these kinases support the expression of the receptors/channels and/or transcriptional factors involved in anti-nociception [51]–[54]. For example, the phosphorylation of MAPK activates transcription factors, such as CREB, which regulates dynorphin gene expression [53], [55]. Our results are consistent with the previously reported results showing that the κ-opioid receptor agonist U 50, 488 activates MAPKs, such as ERK1/2 and p38 MAPK [20], [50]. Furthermore, we propose that the effect of CRP on ERK phosphorylation is mediated by activation of the κ-opioid receptor because the selective opioid receptor antagonist Nor-BNI blocked this effect. We also found that Nor-BNI activates the JNK pathway. These results are consistent with previous results showing that Nor-BNI increases the level of phospho-JNK in the HEK293 cell line [56]. It has been well established that the pharmacological effects of Nor-BNI, such as the antagonistic effect on agonist-induced anti-nociception and intracellular signaling, are long lasting [56], [57] and involve an interaction between κ-opioid receptors and JNK phosphorylation [56]. Therefore, the JNK pathway may play an important role in κ-opioid interacting substrates and may contribute to the long lasting activity of CRP[11].

Based on the results showing that CRP induces the phosphorylation of MAPKs and considering that protein kinase C (PKC) is involved in numerous intracellular signaling pathways in several cellular models [58], we next investigated the role of PKC in CRP-mediated signaling. A previous study by Belcheva et al. (2005) using cultured astrocytes, showed that PKCζ is involved in the activation of MAPKs by opioid agonists [20]. To examine the role of PKCζ in the activation of MAPKs by CRP, we used the PKCζ pseudosubstrate, which selectively inhibits the atypical ζ- and λ- PKC isozymes [35]. Because PKCλ is not expressed in DRGs, the ζ pseudosubstrate should only selectively inhibit PKCζ in these cells. Our results show that PKCζ activation is involved in the CRP-induced increase in phospho-ERK1/2 and JNK in cultured DRG cells; treating these cells with the ζ pseudosubstrate blocked the activation of these kinases by CRP. These results are consistent with the results obtained by Belcheva et al. (2005) [20] that showed U 69,593, a κ-opioid receptor agonist, stimulates the activation of MAPKs *via* PKCζ.

In conclusion, we propose that CRP has a more potent peripheral anti-nociceptive effect under conditions of acute sensitization. The mechanisms responsible for this phenomenon involve increased expression levels and the activation of opioid receptors as well as increases in the cGMP levels, the phosphorylation of MAPKs and the activation of PKCζ in the periphery. These results further elucidate the important peripheral molecular mechanisms involved in pain control and identify interesting therapeutic alternatives for inducing peripheral analgesia.

## Supporting Information

Figure S1 **Time-course for intraplantar injection of prostaglandin E2 (PGE2) in the rat nociceptive threshold.** Pain threshold was obtained in the rat paw pressure test, before (time 0) and 1, 3, 5 and, 8 h after intraplantar injection of PGE2 (100 ng/paw) or saline (control). Data represent mean values ± S.E.M. for six rats per group. * significantly different from baseline, # significantly different from control. Data were analyzed by two-way analysis of variance (ANOVA) with *post-hoc* testing by Tukey.
(TIF)

Figure S2 **Effect of methiodide naloxone (MN) on the local crotalphine (CRP) and opioid receptor agonists-induced anti-nociception.** Pain threshold was obtained in the rat paw pressure test, before (dotted line) and 3 h after intraplantar injection of PGE_2_ (100 ng/paw). CRP (0.6 ng/paw), DAMGO (μ opioid receptor agonist, 5 µg/paw), DPDPE (δ opioid receptor agonist, 20 µg/paw), U-50488 (κ opioid receptor agonist, 10 µg/paw) were injected 2 h after PGE2 administration. NM (1 mg/Kg), were injected by the subcutaneous route 15 minutes before the nociceptive threshold assessment. Data represent mean values ± S.E.M. for five rats per group. * significantly different from baseline (dotted line), # significantly different from control (saline = SAL). Data were analyzed by two-way analysis of variance (ANOVA) with *post-hoc* testing by Tukey.
(TIF)

Figure S3 **Comparative effect between systemic and local injection of crotalphine (CRP) and opioid receptor agonists.** Pain threshold was obtained in the rat paw pressure test, before (dotted line) and 3 h after intraplantar injection of PGE_2_ (100 ng/paw). CRP (0.6 ng), DAMGO (μ opioid receptor agonist, 5 µg), DPDPE (δ opioid receptor agonist, 20 µg), U-50488 (κ opioid receptor agonist, 10 µg) were injected by subcutaneous (systemic) or intraplantar (local) route, 2 h after PGE2 administration. Data represent mean values ± S.E.M. for five rats per group. * significantly different from baseline (dotted line), # significantly different from control (saline = Sal). Data were analyzed by two-way analysis of variance (ANOVA) with *post-hoc* testing by Tukey. The following bars CRP intraplantar, DAMGO intraplantar, DPDPE intraplantar and U 50,488 intraplantar were re-drawn from the Fig. S2, since this experiments were performed in the same day.
(TIF)

Figure S4 **Intraplantar injection of prostaglandin E2 (PGE2) in the rat nociceptive threshold.** Pain threshold was obtained in the rat paw pressure test, before (time 0) and 3 h after intraplantar injection of PGE2 (100 ng/paw) or saline (control). (**A**) values for animals whose tissued were used for mRNA extraction and (**B**) for protein extraction. Data represent mean values ± S.E.M. for 6–8 rats per group. * significantly different from baseline, # significantly different from control. Data were analyzed by two-way analysis of variance (ANOVA) with *post-hoc* testing by Tukey.
(TIF)
