# Supplementary figures and images for: Peripheral Sensitization Increases Opioid Receptor Expression and Activation by Crotalphine in Rats
Source: PLoS One. 2014 Mar 4;9(3):e90576. doi: 10.1371/journal.pone.0090576 (PMC3942445; doi:10.1371/journal.pone.0090576)

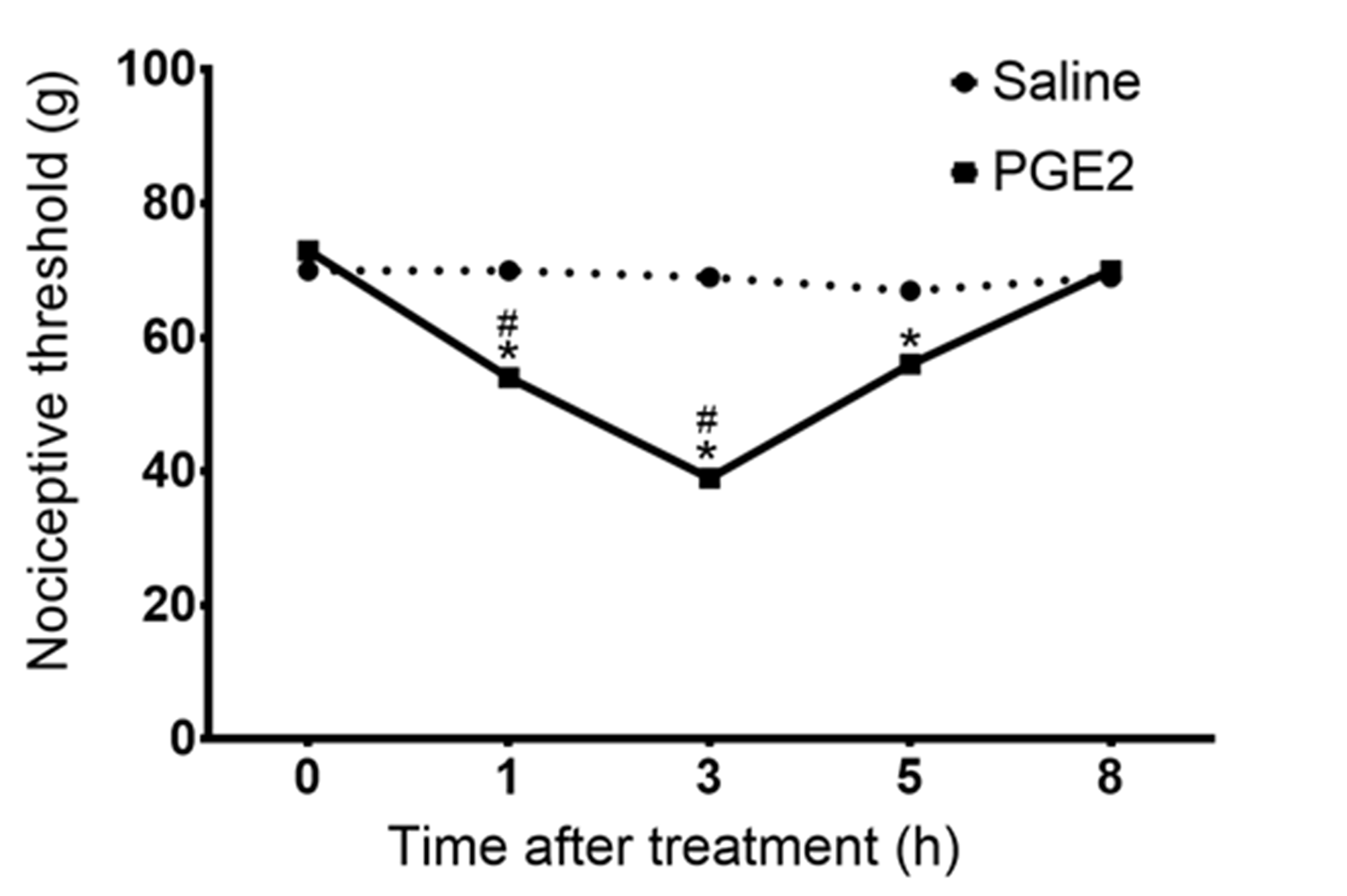

Supplement: Figure S1 — Time-course for intraplantar injection of prostaglandin E2 (PGE2) in the rat nociceptive threshold. Pain threshold was obtained in the rat paw pressure test, before (time 0) and 1, 3, 5 and, 8 h after intraplantar injection of PGE2 (100 ng/paw) or saline (control). Data represent mean values ± S.E.M. for six rats per group. * significantly different from baseline, # significantly different from control. Data were analyzed by two-way analysis of variance (ANOVA) with post-hoc testing by Tukey. (TIF) [file pone.0090576.s001.tif]

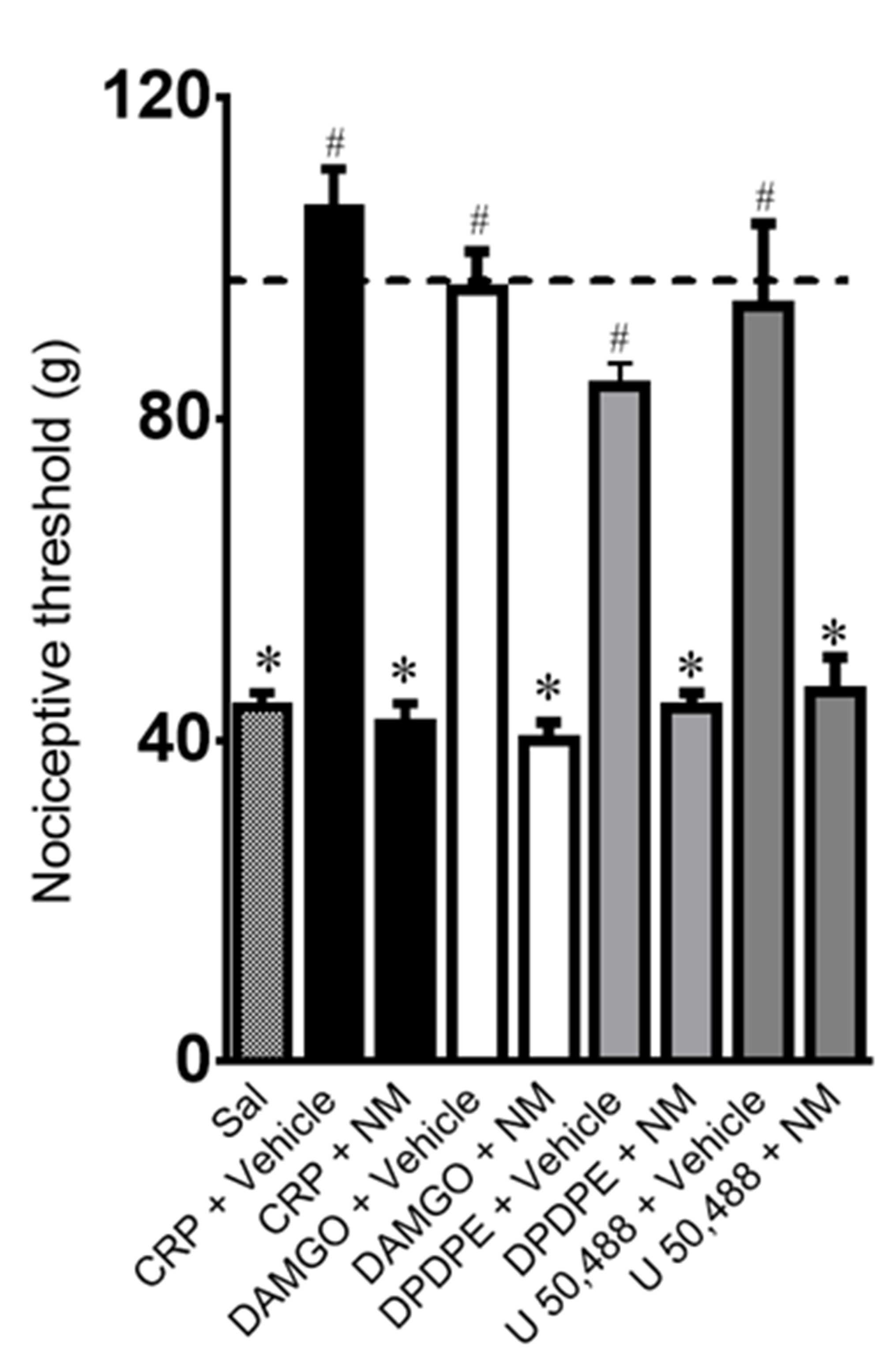

Supplement: Figure S2 — Effect of methiodide naloxone (MN) on the local crotalphine (CRP) and opioid receptor agonists-induced anti-nociception. Pain threshold was obtained in the rat paw pressure test, before (dotted line) and 3 h after intraplantar injection of PGE2 (100 ng/paw). CRP (0.6 ng/paw), DAMGO (μ opioid receptor agonist, 5 µg/paw), DPDPE (δ opioid receptor agonist, 20 µg/paw), U-50488 (κ opioid receptor agonist, 10 µg/paw) were injected 2 h after PGE2 administration. NM (1 mg/Kg), were injected by the subcutaneous route 15 minutes before the nociceptive threshold assessment. Data represent mean values ± S.E.M. for five rats per group. * significantly different from baseline (dotted line), # significantly different from control (saline = SAL). Data were analyzed by two-way analysis of variance (ANOVA) with post-hoc testing by Tukey. (TIF) [file pone.0090576.s002.tif]

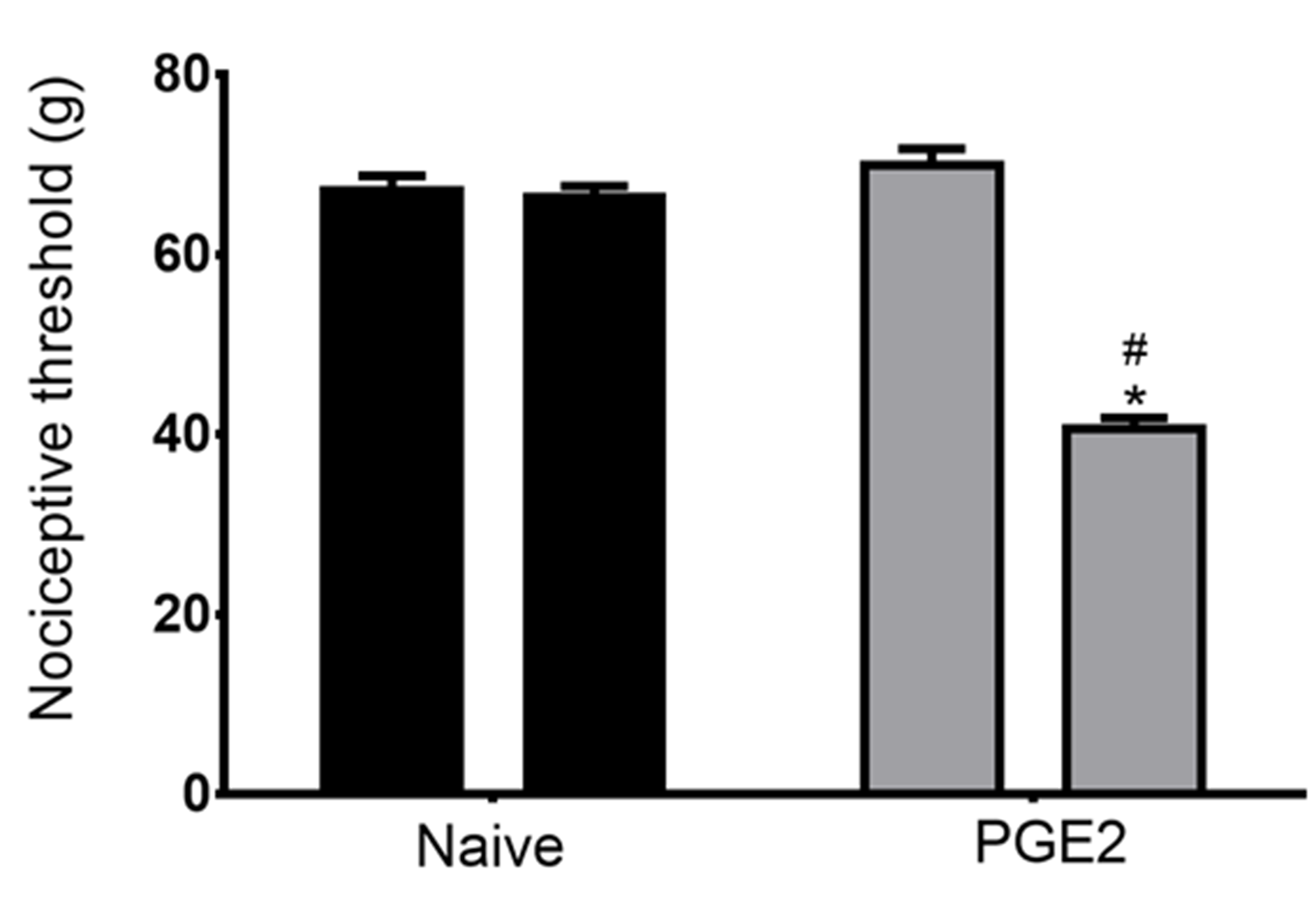

Supplement: Figure S4 — Intraplantar injection of prostaglandin E2 (PGE2) in the rat nociceptive threshold. Pain threshold was obtained in the rat paw pressure test, before (time 0) and 3 h after intraplantar injection of PGE2 (100 ng/paw) or saline (control). (A) values for animals whose tissued were used for mRNA extraction and (B) for protein extraction. Data represent mean values ± S.E.M. for 6–8 rats per group. * significantly different from baseline, # significantly different from control. Data were analyzed by two-way analysis of variance (ANOVA) with post-hoc testing by Tukey. (TIF) [file pone.0090576.s004.tif]
